# Poisoning by Caterpillars

**Published:** 1898-09

**Authors:** 


POISONING BY CATERPILLARS. Girard, a veterinary surgeon at Barre, Vt., has observed numerous fatal cases of poisoning in ducks after eating caterpillars, notably those of *pieris brassicæ*, the large, white cabbage butterfly. About six hours after eating these larvæ poisoning became evident, diarrhœa, and staggering gait, followed by dyspnœa and ultimately by death.

Autopsy showed the essential lesions to indicate inflammation of the digestive tube. It is probable that these symptoms are caused by the inflammation produced in the alimentary canal by the minute hairs with which this caterpillar is covered. It has been noticed that chickens invariably refused the larvæ of *P. brassicæ*, although they greedily eat the smooth larvæ of the various noctura.—*Popular Science News.*

